# In everybody’s interest but no one’s assigned responsibility: midwives’ thoughts and experiences of preventive work for men’s sexual and reproductive health and rights within primary care

**DOI:** 10.1186/s12889-019-7792-z

**Published:** 2019-10-30

**Authors:** Maria Grandahl, Maja Bodin, Jenny Stern

**Affiliations:** 1 Department of Women’s and Children’s Health, Uppsala University, Akademiska sjukhuset, SE-751 85 Uppsala, Sweden; 2 Sophiahemmet University, Box 5605, SE-114 86 Stockholm, Sweden

**Keywords:** Equal health, Gender norms, Health care providers, Health promotion, Men, Midwives, Sexual and reproductive health and rights, Social model of health

## Abstract

**Background:**

Sexual and reproductive health and rights (SRHR) have historically been regarded as a woman’s issue. It is likely that these gender norms also hinder health care providers from perceiving boys and men as health care recipients, especially within the area of SRHR. The aim of this study was to explore midwives’ thoughts and experiences regarding preventive work for men’s sexual and reproductive health and rights in the primary care setting.

**Methods:**

An exploratory qualitative study. Five focus group interviews, including 4–5 participants in each group, were conducted with 22 midwives aged 31–64, who worked with reproductive, perinatal and sexual health within primary care. Data were analysed by latent content analysis.

**Results:**

One overall theme emerged, in everybody’s interest, but no one’s assigned responsibility, and three sub-themes: (i) organisational aspects create obstacles, (ii) mixed views on the midwife’s role and responsibility, and (iii) beliefs about men and women: same, but different.

**Conclusions:**

Midwives believed that preventive work for men’s sexual and reproductive health and rights was in everybody’s interest, but no one’s assigned responsibility. To improve men’s access to sexual and reproductive health care, actions are needed from the state, the health care system and health care providers.

## Background

One of the main objectives of the European policy for health and well-being is to improve health for all and reduce health inequalities [1]. The World Health Organization (WHO) recognise sexual and reproductive health and rights (SRHR) as one of the most important areas to strengthen and protect to ensure physical and mental health. SRHR have historically been regarded as a woman’s issue. Even though men’s perspectives are gaining more interest and one of the Sustainable Development Goal targets is to ensure universal access to sexual and reproductive health care [2], it remains less explored and men are still underrepresented in the social debate and health care as well as in research when it comes to SRHR [3].

According to the Social Model of Health (SMH), individuals’ health is affected not only by biological and lifestyle factors but also social factors such as gender, education, health services, political decisions and health policies [4]. For example, traditional norms of masculinity have been suggested to promote ideals, which means putting one’s health at risk as well as hindering boys and men from perceiving themselves as recipients of care [5–7]. Consequently, men complying with these masculinity ideals are less likely to engage in health promoting behaviours [8]. Several studies have found that men use sexual and reproductive services, e.g. STI testing, to a lower extent than women [9–14] and have less knowledge about how to access such services [15, 16]. As has been shown to be the case with other normative attitudes [17, 18], it is likely that masculinity norms also hinder health care providers (HCPs) from perceiving boys and men as health care recipients and delivering adequate care, especially within the area of SRHR.

Sweden is often portrayed as a forerunner regarding gender equality, and the Swedish Health Care Act (2017:30) states that the goal of the health care is good health and care on equal terms for the entire population. However, without a national strategy for SRHR, the health care provision for SRHR is organised in different ways in different regions. Local guidelines differ, both with respect to target group and prioritised areas. For adolescents and young adults, all regions provide youth clinics for all genders, but only 13% of visitors are male [19]. For adults, the main arena for SRHR is midwifery clinics, which are mainly directed towards girls and women. Men’s clinics are few but can be found in larger cities.

In the absence of guidelines for reproductive health provision for men, it is important to investigate HCPs point of view on this topic. The aim of this study was therefore to explore the thoughts and experiences of midwives working in the primary care setting concerning their preventive work for men’s sexual and reproductive health and rights.

## Methods

### Design

The study had an exploratory qualitative design using focus group interviews (FGIs). We used the Social Model of Health to discuss our findings (see Introduction). The study was conducted as part of a larger study on health care providers’ adoption of a health-promoting tool for SRHR [20].

### Setting

In Sweden, national laws regulate health care in general, but health care provision is organised autonomously in 21 regions. Reproductive, perinatal and sexual health is the area of competence for registered nurse-midwives (RNMs) and obstetricians/gynaecologists. Contraceptive counselling is mainly offered by RNMs within the primary health care system. The present study was conducted in one region in mid Sweden that covers both rural and urban areas. In 2014, the region had 345,000 inhabitants and 68 RNMs working at 21 midwifery clinics and 9 youth clinics. Henceforth, RNMs will be referred to as midwives, to facilitate the reading of the paper.

### Data collection

Data were collected in spring 2014. The procedure has previously been described by Stern et al. [20]. Primary care midwives working with SRHR in one region in mid Sweden were invited to participate. Out of 53 eligible, 22 midwives volunteered to participate in the focus group interviews. Background characteristics of the participants are presented in Table 1. Five FGIs with 4–5 participants per group were conducted in conference rooms at the university or the midwifery clinic. Each group included participants of different ages, with varying work experience, from public and private clinics. A moderator (female registered nurse or midwife) led the interviews based on an interview guide, and an observer (female registered nurse or midwife) kept track that all topics were covered. The interview guide was previously presented in Stern et al. [20] and comprised questions about midwives’ 1) adoption of a health promoting tool for reproductive health, used during contraceptive counselling as well as 2) thoughts and experiences of preventive work for men’s sexual and reproductive health. Follow-up questions were asked to clarify or elaborate statements and invite other participants to comment. The interviews were recorded with an average length of 91 min (range 64–118 min), and transcribed verbatim. Participants received a cinema ticket for their participation.

**Table 1:** Description of included midwives (*n* = 22)

Characteristics	mean (SD)
Age, years	51.5 (11.0)
Work experience from contraceptive counselling, years	13.3 (11.2)
No. of contraceptive counselling sessions/week	15.4 (6.9)
	n (%)
Workplace	
Public	16 (73)
Private	6 (27)
Type of clinic	
Midwifery clinic	17 (77)
Youth clinic	4 (18)

### Data analysis

To analyse the data, we used qualitative latent content analysis as described by Burnard et al. [21]. This is an inductive approach where the actual data is used to derive the structure of analysis. First, transcripts were read several times to get an overview. The data were then coded based on the aim, with notes made in the margins to summarise the relevant data. All codes were then reviewed, and duplicates deleted. Thereafter, we started looking for overlaps and similarities among codes. During this process, the codes were collated and compiled into categories and finally into sub-themes and themes. The themes describe the latent content of the analysis from the interviews [22]. All steps of the analysis involved a back and forth movement to the text to ensure validity and were conducted by all of the authors. We did not use any specific software in the analysis. However, we used Excel© to manage the data. Examples of the analytical process are presented in Table 2.

**Table 2:** Example of the analytical process

Interview transcript	Initial coding	Category	Sub-theme	Theme
‘Well, I don’t want to close any doors. I think it’s good that women and men can come to the same clinic, you shouldn’t divide it like “you go there and you go there”, as if we are that different’. [FG1]	Good to not divide women and men	Men and women’s needs are intertwined	Beliefs about men and women: same, but different	In everybody’s interest, but no one’s assigned responsibility

## Results

One theme and three sub-themes emerged from the analyses of the FGIs exploring midwives’ thoughts and experiences regarding preventive work for men’s sexual and reproductive health and rights (Table 3).

**Table 3:** Overview of the results

Theme	Sub-themes	Categories
In everybody’s interest, but no one’s assigned responsibility	Organisational aspects create obstacles	Men have nowhere to turn
	Beliefs about men and women: same, but different	Different health seeking behaviour, interest and needs among men than women
		Men and women’s needs are intertwined
	Mixed views on the midwife’s role and responsibility	Strategies to involve men
		Being hesitant to provide consultation to men

### Theme: in everybody’s interest, but no one’s assigned responsibility

#### Organisational aspects create obstacles

##### Men have nowhere to turn

There were organisational conditions that hindered midwives’ opportunities to deliver sexual and reproductive care to men. Midwives expressed concerns about men having nowhere to turn to with their questions about reproduction, while women had an obvious entry to the health care system through contraceptive counselling.

*‘But girls are called for pap smear tests and meet midwives where they get some contacts and... have another contact with health care providers, perhaps in a different way than what guys have.[FG5]*

*‘It is no wonder that more is put on the women, because where will they [men] go?! It might be a 24-year-old guy who has no ailments or anything but who has many thoughts, where should he turn with those? It's not so obvious who he should call’. [FG1]*

Men were perceived as hard to reach, and they seldom showed up at the midwifery clinic following adolescence. There was little confidence that parents would take on the responsibility to educate their children, as it was expressed that *‘parents are just getting more and more stressed today and do not have the energy’* [FG3].

Documentation was another obstacle. In the current patient record system, it was not possible during consultations with couples to write notes in the partner’s record if the woman had made the appointment. Neither was there any time allocated during the appointment, specifically for men. Midwives had to come up with their own solutions of how to make time for them. This was not an easy task, and these visits did not generate any financial compensation.

#### Beliefs about men and women: same, but different

##### Different health seeking behaviour, interest and needs among men than women

The needs, rights and responsibilities of men were mostly discussed in the context of being the partner of a woman. For example, it was emphasised that men should be concerned about contraception, but that it was the woman’s choice if she wanted to bring her partner to contraceptive counselling or not.

There was a strong perception that men did not seek health care very often, unless it was for severe symptoms or conditions. However, midwives had noted that more and more young men attended STI-testing and showed interest and openness to discuss sexual health. Some midwives believed that it was a man’s right to get information, and that it was important to expand their knowledge about reproduction.

*‘Surprisingly often, when we meet guys nowadays […] they don’t really know how someone becomes pregnant and common simple basic knowledge’.*

*‘So, we have started to draw the menstrual cycle [on paper], which they might not need, but I believe it’s good that they become aware of how it works with guys and sperm…’*[FG3]

The midwives also found it important to give young men the opportunity to talk about emotions and concerns, and to inform them about the juridical consequences of having unprotected sex (i.e. being responsible for paying child support). Men should take responsibility for their sexual life and not only trust the female partner to take care of the contraception. However, the midwives wanted men to be involved “just enough” [FG5]; he should not be “the fifth wheel” [FG1] during the visit, but if he was too involved then midwives started to suspect a controlling behaviour and intimate partner violence. Still, most men were perceived as taking a step back and letting the woman be in focus; consequently, letting the woman take major responsibility for their joint future.

Midwives experienced that women and men had equally many questions, although different ways of thinking and different needs when it came to sexual and reproductive health. One of the responses was *‘Guys are under pressure in a different way than girls, especially when you’re young and with sexuality and all that’* [FG3]. Men were perceived as worrying quite a lot about erectile dysfunction and the consequences of unprotected sex and STIs. But there was also a perception that young men did not think about the future, only about the present, and would therefore not see themselves as target group for reproductive health information.

Age was perceived as an important factor in the equation. Some midwives believed that guys who were still in high school or who attended youth clinics were too young to talk to; they were “not there yet”. Others reasoned that individual maturity and experience mattered more. Thoughts around fertility would be relevant to those who have found a partner or had experienced an unplanned pregnancy.

*‘I think it’s really great during contraceptive counselling, [or] with guys who are there for STI-testing, in whatever age they are, you can actually do this… you can bring this up and make them think […] it doesn’t matter if they are 18 or 40’. [FG1]*

##### Men and women’s needs are intertwined

The midwives also stressed that parents-to-be should be viewed as a unit, meaning that *‘he also becomes pregnant or how do I say it*’ [FG5]. In exceptional cases, the midwives would address the man’s needs during pregnancy, with the motivation that it would benefit the child to have a healthy father.

Midwives believed it would be better for all parties if preventive work for men’s SRHR were improved since women and men’s sexual and reproductive health are intertwined. A start could be to encourage couples to talk to each other about SRHR and to share the cost of contraceptives. This would contribute to men feeling more involved in the decision-making and create SRHR as a common interest for both partners.

*‘If they are together with a female partner... it is interesting for them too; they should be able to discuss this. If they don’t know that women's fertility goes down a lot at age thirty-five. I mean we need to talk about it in our relationship or about what we think because it's tragic for both if they don't have children, it's really bad for both’. [FG5]*

#### Mixed views on the midwife’s role and responsibility

When asked about their own role and responsibility regarding preventive work for men’s SRHR, the opinions differed. There were midwives who already had experience from counselling men, for example, about STIs and erectile dysfunction. They found it interesting to talk about reproduction and health with men. Some midwives were very positive towards implementing counselling for men into their work, and they saw it as exciting, fun and natural part of their current assignments.

*‘Well, I don’t want to close any doors. I think it’s good that women and men can come to the same clinic, you shouldn’t divide it like “you go there and you go there”, as if we are that different’. [FG1]*

The couple’s consultation session was seen by some as an opening for future individual conversations with men. Other participants believed that it was difficult to include the partner during joint contraceptive counselling. This resulted in men’s health usually being addressed on opportunistic basis rather than planned.

##### Strategies to involve men

Experiences of conversations with men came about in quite different ways. Several midwives believed that there was no need to be too detailed with men and thought that just asking a few questions could “plant a seed” in some men’s minds. Some agreed that it was important not to be too moralistic and that everyone should feel safe and be in focus regardless of gender. Others warned men that they could be fooled by their partner and get in trouble if they did not use protection. Talking about reproductive life plans was considered as being less sensitive than talking about someone’s genitals, but to talk about it with couples was perceived as a delicate matter. Therefore, midwives believed that it would be suitable to talk to young men about sexual and reproductive health at youth clinics or in connection with STI-testing, since these were the few arenas and opportunities where they met men regularly.

*‘Yes, in nine cases out of ten, guys come to us to undergo chlamydia tests. They just want to get help ... get in quickly and take the test and be told how it works and then goodbye .... I think more if you could ... talk to guys at youth clinics’. [FG3]*

They suggested that other arenas for sexual and reproductive counselling were needed for men, in addition to or instead of midwifery clinics. They indicated that venereology or urology clinics, or specific clinics for men where male HCPs with training in andrology worked, were relevant arenas. It was also argued that teachers or school nurses should take on the responsibility to educate young men. In Sweden, school nurses are responsible for having a general health consultation with pupils, which was viewed as a good opportunity to reach boys with information about sexual and reproductive health. Sports clubs could also be involved. It was also suggested that men could be reached at postnatal check-ups and at infertility clinics.

##### Being hesitant to provide consultation to men

There were also midwives who were more reluctant to providing consultation to men. It was not evident to them that men should turn to midwives, partly since a pregnancy is about the female body. They motivated their standpoints by saying *‘it’s important, but it’s not our task’* [FG5] or *‘there are billions of things we have to think about giving her and explaining and informing and so on, so I feel I don’t have the energy to inform guys or include them in this…’* [FG3]. Others were indifferent, and they neither encouraged nor discouraged men from contributing and participating.

If midwives were to provide consultation to men in the future, they expressed a need for further education in andrology since many midwives lacked enough knowledge about male reproduction. Lack of knowledge caused insecurity in the counselling situation. To meet the needs of men with regard to sexual and reproductive care in the future, education geared towards midwives would need to be revised or a new profession would have to be invented.

*‘There was a guy who wanted to show me some kind of rash [on his penis] and I just “No!” I kind of panicked […] Well, then I felt, I am not knowledgeable about this’ [FG5]*

*‘But I am a midwife and don’t know much about men's fertility! It is awful really. You are at an antenatal clinic, it feels like one should know this’. [FG1]*

## Discussion

The midwives interviewed in this study believed that preventive work for men’s sexual and reproductive health and rights was in everybody’s interest, but no one’s assigned responsibility. Men were described as being let down by society and having nowhere to turn to. However, the participants expressed contradictory opinions as to whether men fell under the midwife’s responsibility or not and described organisational factors that constituted obstacles to delivering this type of care. Men were perceived as having both equal and different needs and interests than women.

Looking at the results in the perspective of SMH, we can identify several social factors that influence men’s SRHR; the organisation of health care services, national health policies as well as gender norms [4–7]. One main finding was that men, especially above the age where they could visit youth clinics, were regarded as falling through the cracks, which has also been identified in other contexts [23]. This implicates the need for national strategies for SRHR, including allocation of responsibilities, as well as regional guidelines that include men as a target group for SRHR. Further, in line with previous research [24, 25], organisational factors must be adjusted to enable preventive work for men’s SRHR, for example, adaption of electronic records and economic compensation systems for this kind of visits.

Another main finding was the disagreement among midwives regarding whether preventive work for men’s SRHR is their responsibility or not. In Sweden, the National Board of Health and Welfare define the area of competence for midwives, and midwives’ role and responsibility for men’s SRHR evidently need to be clarified in the competence description.

The interviews also revealed low knowledge about SRHR for men; moreover, as stated by the included midwives, it is important with further education about male reproduction among HCPs. The question of who is the most appropriate service provider probably differs between contexts and health care systems [26–28], but regardless of profession, there is a need for clear definitions and standards for clinical care as well as adequate knowledge in the topic [10]. As long as the question of responsibility is open for interpretation, we believe preventive work for men’s SRHR will continue to be provided on a diminutive and opportunistic basis. It becomes clear from the interviews that men today do not receive equal care; rather, the provision and quality of care is dependent on the individual midwives’ interest in and beliefs about men’s SRHR.

The participants both identified and reproduced gender norms for procreative responsibility. Men were most often talked about in relation to a woman and seldom as reproductive beings in their own right. This can be regarded both a consequence and reproducing mechanism of the gender norms that permeate many societies. The assumption that the woman carries the main responsibility for reproduction reflects on all levels in the SMH [4], and health care services are no exception. Men’s limited health seeking behaviour has been problematised within research [8–13] and in Sweden, less than 15% of all adolescents attending youth health clinics for SRHR counselling and STI testing are men. This behaviour is unlikely to change by itself if there is no obvious arena for men to seek care throughout life. Recent studies have highlighted men’s interest in SRHR and their wish to receive more information from HCPs [29–32]. Our results illustrated a gap in health care provision, between preventive work at youth clinics and obstetric care/infertility treatment, which is more pronounced for men than women. This reinforces the norm that men’s fertility is a non-issue, and rather seen as something self-evident [7, 33]. This phenomenon is especially pronounced in sexual and reproductive health care since men’s sexuality, in contrast to women’s, is seen as something that just works well by itself [34]. However, men and women’s needs were viewed as intertwined, and the midwives revealed several strategies to reach men. They emphasised that schools were suitable arenas in order to reach adolescents regardless of gender, socioeconomic status, and country of birth or other social factors [4]. Reducing inequalities, promoting gender equality and good sexual- and reproductive health care on equal terms are in line with UNs Sustainable Development Goals and the national public health goals in Sweden [2]. To start early and promote SRHR regardless of gender has previously been discussed [30, 32] and might be one way to improve and preserve fertility and reproductive health for the individual- as well as the public health.

This study is based on FGIs with midwives in Sweden. The focus group method is recommended for exploring people’s knowledge and experiences [35]. The discussions were lively, where contradictory views were expressed which indicates that participants perceived the environment as safe, thereby, strengthening the validity of the data. The study follows Standards for Reporting Qualitative Research and is reported according to COREQ Checklist (Additional file 1) [36, 37]. The analysis process was systematic and rigorous, all data transcripts were thoroughly analysed, and the main findings as well as contrary findings have been presented in the results section. To check for validity and to avoid lone researcher bias, the researchers individually read the transcripts to identify categories. All researchers took part in discussing the categories and themes until a consensus was reached. Often portrayed as one of the most equal countries, the Swedish setting is interesting as a best-case scenario.

We continued with data collection until information power, i.e. adequate information on the topic, was achieved. The more information the sample holds, the lower number of participants is needed [38]. Thus, we consider the sample size adequate for gaining sufficient data for this specific study. The topic was extensively discussed, and the participants generously shared their thoughts and experiences, which contributed to rich data material. The participants represented a wide range of ages, years of work experience and workplaces, but not all midwives had experience from working preventively with men’s reproductive health. However, the narratives largely came to represent attitudes and beliefs relating to the matter. Participants were recruited from one out of 21 regions; hence, it is theoretically possible that there are regional variations regarding opinions on preventive work for men’s SRHR. Nevertheless, the aim of this qualitative study was not to generalise but rather explore midwives’ opinions in a setting with no national guidelines for preventive SRHR for men.

## Conclusions

Midwives working within primary care believed that preventive work for men’s sexual and reproductive health and rights is in everybody’s interest, but no one’s assigned responsibility. As long as there are no guidelines or self-evident arenas for men to seek advice regarding reproductive health, it is likely that care for men will continue to be delivered on arbitrary basis. To improve men’s sexual and reproductive health, actions need to be taken on policy and community level.

## Supplementary information

**Additional file 1.** COREQ Checklist, Standards for Reporting Qualitative Research Description of data.

## Data Availability

The datasets generated and analysed during the current study are not publicly available due to the risk of identifying participants but are available from the corresponding author upon reasonable request.

## Abbrevations

FGI: Focus group interview
HCPs: Health care providers
RNMs: Registered nurse-midwives
SMH: Social model of health
SRHR: Sexual and reproductive health and rights
STI: Sexually transmitted infection

## References

[1] WHO. Health 2020: the European policy for health and well-being http://www.euro.who.int/en/health-topics/health-policy/health-2020-the-european-policy-for-health-and-well-being/about-health-2020/strategic-objectives. Accessed 19 Jan 2019.

[2] UN. Sustainable Development Goals https://www.un.org/sustainabledevelopment/sustainable-development-goals/. Accessed 19 Jan 2019.

[3] Inhorn M, Tjørnhøj-Thomsen T, Goldberg H, Mosegaard M la C: Reconceiving the Second Sex. Men, Masculinity, and Reproduction New York: Berghahn Books; 2009.

[4] WHO. European strategies for tackling social inequities in health: Levelling up Part 2 http://www.euro.who.int/__data/assets/pdf_file/0018/103824/E89384.pdf. Accessed 19 Jan 2019.

[5] Bender SS, Fulbright YK (2013). Content analysis: a review of perceived barriers to sexual and reproductive health services by young people. Eur J Contracept Reprod Health Care.

[6] Lindberg C, Lewis-Spruill C, Crownover R (2006). Barriers to sexual and reproductive health care: urban male adolescents speak out. Issues Compr Pediatr Nurs.

[7] Courtenay WH, Keeling RP (2000). Men, gender, and health: toward an interdisciplinary approach. J Am Coll Heal.

[8] Mahalik JR, Burns SM, Syzdek M (2007). Masculinity and perceived normative health behaviors as predictors of men's health behaviors. Soc Sci Med.

[9] Marcell AV, Gibbs SE, Pilgrim NA, Page KR, Arrington-Sanders R, Jennings JM, Loosier PS, Dittus PJ (2018). Sexual and reproductive health care receipt among young males aged 15-24. J Adolesc Health.

[10] Kalmuss D, Tatum C (2007). Patterns of men's use of sexual and reproductive health services. Perspect Sex Reprod Health.

[11] Pazol K, Robbins CL, Black LI, Ahrens KA, Daniels K, Chandra A, Vahratian A, Gavin LE (2017). Receipt of selected preventive health Services for Women and men of reproductive age - United States, 2011-2013. MMWR Surveill Summ.

[12] Marcell AV, Bell DL, Lindberg LD, Takruri A (2010). Prevalence of sexually transmitted infection/human immunodeficiency virus counseling services received by teen males, 1995-2002. J Adolesc Health.

[13] Lewis R, Tanton C, Mercer CH, Mitchell KR, Palmer M, Macdowall W, Wellings K (2017). Heterosexual practices among young people in Britain: evidence from three National Surveys of sexual attitudes and lifestyles. J Adolesc Health.

[14] Public Health Agency of Sweden [Folkhälsomyndigheten]: Chlamydiainfektion. https://www.folkhalsomyndigheten.se/folkhalsorapportering-statistik/statistikdatabaser-och-visualisering/sjukdomsstatistik/klamydiainfektion/. Accessed 19 Jan 2019.

[15] Newton-Levinson A, Leichliter JS, Chandra-Mouli V (2016). Sexually transmitted infection Services for Adolescents and Youth in low- and middle-income countries: perceived and experienced barriers to accessing care. J Adolesc Health.

[16] Bersamin M, Fisher DA, Marcell AV, Finan LJ (2017). Reproductive health services: barriers to use among college students. J Community Health.

[17] Jonas K, Crutzen R, van den Borne B, Reddy P (2017). Healthcare workers' behaviors and personal determinants associated with providing adequate sexual and reproductive healthcare services in sub-Saharan Africa: a systematic review. BMC Pregnancy Childbirth.

[18] Godia PM, Olenja JM, Lavussa JA, Quinney D, Hofman JJ, van den Broek N (2013). Sexual reproductive health service provision to young people in Kenya; health service providers' experiences. BMC Health Serv Res.

[19] Public Health Agency of Sweden [Folkhälsomyndigheten]: Ungdomar och sexualitet 2014/2015. http://www.folkhalsomyndigheten.se/documents/sexualitethalsa/Uungdomsbarametern-2014.pdf. .

[20] Stern J, Bodin M, Grandahl M, Segeblad B, Axen L, Larsson M, Tyden T (2015). Midwives' adoption of the reproductive life plan in contraceptive counselling: a mixed methods study. Hum Reprod.

[21] Burnard P, Gill P, Stewart K, Treasure E, Chadwick B (2008). Analysing and presenting qualitative data. Br Dent J.

[22] Graneheim UH, Lindgren BM, Lundman B (2017). Methodological challenges in qualitative content analysis: a discussion paper. Nurse Educ Today.

[23] Kramer H, Lehmann J, Klapp C, Layer C, Mais A, Kriwy P (2018). Is there also a gynecologist for men? : A randomised controlled trial of AGGF information sessions in schools as a bridge to the urologist's consultations with boys. Urologe A.

[24] Jonas K, Crutzen R, Krumeich A, Roman N, van den Borne B, Reddy P (2018). Healthcare workers' beliefs, motivations and behaviours affecting adequate provision of sexual and reproductive healthcare services to adolescents in Cape Town, South Africa: a qualitative study. BMC Health Serv Res.

[25] Collyer A, Bourke S, Temple-Smith M (2018). General practitioners' perspectives on promoting sexual health to young men. Aust J Gen Pract.

[26] Marcell AV, Burstein GR, Committee On A: Sexual and Reproductive Health Care Services in the Pediatric Setting. Pediatrics. 2017;140(5):1–13. 10.1542/peds.2017-2858 29061870

[27] Tabong PT, Maya ET, Adda-Balinia T, Kusi-Appouh D, Birungi H, Tabsoba P, Adongo PB (2018). Acceptability and stakeholders perspectives on feasibility of using trained psychologists and health workers to deliver school-based sexual and reproductive health services to adolescents in urban Accra, Ghana. Reprod Health.

[28] French RS, Geary R, Jones K, Glasier A, Mercer CH, Datta J, Macdowall W, Palmer M, Johnson AM, Wellings K. Where do women and men in Britain obtain contraception? Findings from the third National Survey of sexual attitudes and lifestyles (Natsal-3). BMJ Sex Reprod Health. 2018;44(1):16–26. 10.1136/jfprhc-2017-101728 PMC6283328 29103003

[29] Daumler D, Chan P, Lo KC, Takefman J, Zelkowitz P (2016). Men's knowledge of their own fertility: a population-based survey examining the awareness of factors that are associated with male infertility. Hum Reprod.

[30] Ekstrand Ragnar M, Grandahl M, Stern J, Mattebo M (2018). Important but far away: adolescents' beliefs, awareness and experiences of fertility and preconception health. Eur J Contracept Reprod Health Care.

[31] Bodin M, Tyden T, Kall L, Larsson M (2018). Can reproductive life plan-based counselling increase men's fertility awareness?. Ups J Med Sci.

[32] Grandahl M, Neveus T, Dalianis T, Larsson M, Tyden T (2019). Stenhammar C: 'I also want to be vaccinated!' - adolescent boys' awareness and thoughts, perceived benefits, information sources, and intention to be vaccinated against human papillomavirus (HPV). Hum Vaccin Immunother.

[33] Sylvest R, Koert E, Vittrup I, Birch Petersen K, Hvidman HW, Hald F, Schmidt L (2018). Men's expectations and experiences of fertility awareness assessment and counseling. Acta Obstet Gynecol Scand.

[34] Public Health Agency of Sweden [Folkhälsomyndigheten]: Mäns sexualitet och reproduktiva hälsa. https://www.folkhalsomyndigheten.se/publicerat-material/publikationsarkiv/m/mans-sexualitet-och-reproduktiva-halsa/. Accessed 19 Jan 2019.

[35] O'Brien BC, Harris IB, Beckman TJ, Reed DA, Cook DA (2014). Standards for reporting qualitative research: a synthesis of recommendations. Acad Med.

[36] Kitzinger J (1995). Qualitative research. Introducing focus groups. BMJ..

[37] Tong A, Sainsbury P, Craig J (2007). Consolidated criteria for reporting qualitative research (COREQ): a 32-item checklist for interviews and focus groups. Int J Qual Health Care.

[38] Malterud K, Siersma VD, Guassora AD. Sample Size in Qualitative Interview Studies: Guided by Information Power Qual Health Res 2015. Epub 2015/11/29. doi: 10.1177/1049732315617444. 10.1177/1049732315617444 26613970

